# Reviews and Bibliographical Notices

**Published:** 1882-04

**Authors:** 


### REVIEWS AND BIBLIOGRAPHICAL NOTICES.

Diseases of Women: Including their Pathology, Causation, Symptoms, Diagnosis, and Treatment. A Manual for Students and Practitioners. By Arthur W. Edis, M.D. (Lond.), F.R.C.P., M.R.C.S., etc. With one hundred and forty-eight illustrations. Philadelphia. Henry C. Lea's Son & Co. 1882. Pp. 576. This manual of the diseases of women, for "the student and junior practitioner," is mainly a compilation from the standard larger works on the subject, although the author gives abundant evidence throughout the volume of having had considerable practical experience. Chapter II. deals with the means and methods of physical diagnosis in uterine diseases. As in nearly all other text-books, Fergusson's tubular speculum is figured, recommended, and over a page devoted to a description of the instrument and the method of using it. It need hardly be said, however, that no gynecologist in this country ever uses either Fergusson's speculum, or Cusco's, which, with its modifications, takes up a page and a half more. As objects of interest connected with the history of the development of gynecology, Fergusson's and Cusco's specula have their value; but in practice they have been long since superseded. The instructions for the introduction of Sims' speculum are rather confused. Goodell's speculum—the best of the bivalves, and Nott's, Erich's or Emmet's self-retaining modifications of Sims', are not referred to, while Neugebauer's, which is just a little less useful than Cusco's, is figured and fully described.

Laminaria tents are preferred to those of sponge, which is not in agreement with the experience of most American practitioners. Tupelo tents are referred to, but the author has evidently never seen or used them. The dangers from the use of tents are very much exaggerated. According to Dr. Edis, "the dangers inseparable from the employment of tents to dilate the cervix should deter any but those having special experience in gynecology from resorting to them." Gynecological specialists ought to favor this doctrine, as it would leave very few patients for the general practitioner to treat.

In Chapters IV., V., VI., and VII., displacements of the uterus are considered. The diagram on page 62, showing the normal position of the uterus, with the accompanying description, is anatomically incorrect. The canal of the uterus is almost parallel to the perpendicular axis of the body, instead of being in the normal degree of anteflexion which B. S. Schultze and Fritsch have demonstrated to exist. The so-called perineal body is a pyramid whose apex reaches nearly to Douglas' pouch—a thing that never existed in nature.

The Hodge lever pessary, figured on page 72, is turned upside down, probably a proof-reader's error. The diagram of the Hodge *in situ*, on the next page, represents the pessary as being hung up on the retro-curved cervix. It certainly does not indicate the manner in which the uterus is supported by the pessary.

Dr. Edis rightly condemns those absurd contrivances which are supposed to keep the uterus in position by over-filling the vagina. Such are the old-fashioned oval or globular box-wood pessary, and the large, circular, disk-shaped pessary. In the February number of this journal a case is reported by Dr. Reeves Jackson, in which a globular pessary had been retained in the vagina for thirty-five years, and which finally required somewhat of a surgical operation for its removal. The objections against Zwanck's and Duncan's stem and disk pessary are also pointed out. On the whole, Dr. Edis' remarks on pessaries are sensible, although we think he does not entirely appreciate their correct mechanism. The author evidently favors the use of intra-uterine stem-pessaries in flexions.

In Chapter XIII., granular and cystic degeneration of the cervix receive judicious treatment, and laceration of the cervix is more fully and comprehensively treated than in any transatlantic work of which we have any knowledge. The sutures in Fig. 110, after Galabin, are incorrectly placed. They should not pass through the entire thickness of the denuded tissue, but only deep enough to bring the vaginal borders of the denudation into accurate contact.

After all the thorough work that has of late years been done by British investigators on the diseases of the ovaries, it would be a marvel indeed if an English author failed to give a satisfactory account of this somewhat difficult subject. Our anticipations are fully realized, for Chapters XVIII., XIX., XX., and XXI., give as complete an account of the diseases of these organs as can be found in any text-book. Twenty-two pages are devoted to the diagnosis of abdominal tumors, and the subject is thoroughly gone over.

The remaining chapters treat of the other diseases and malformations of the genital organs not referred to above, in a clear and comprehensive manner. As a whole the book may be considered as fully abreast of English gynecological practice. The advances made in late years by American and German observers are not noticed with sufficient fulness to render the book of much value as a work of reference for the physician who desires to know the latest additions to our knowledge. In this respect the excellent "Student's Guide" to gynecology of Dr. Galabin has not yet been excelled, although the latter does not cover the ground so thoroughly as the work under review.

While in the pathological portions the work is deficient, it is fuller of the more practical details of diagnosis and treatment than any other work with which we are acquainted. The mechanical execution of the book —paper, binding, and press-work—is excellent. The engraving of a number of the wood-cuts is, however, not so well executed.

*Transactions of the American Medical Association.* Vol. 32, 1881.

Among the more important papers in this volume of the transactions are the novel views of Dr. Hunter McGuire, of Richmond, on operative interference in gunshot wounds of the abdomen; the clinical investigation into the influence of iodide of potassium in the causation of Bright's disease, by Dr. I. E. Atkinson, of Baltimore; the inquiry into the zymotic nature of croupous pneumonia, by Dr. D. W. Prentiss, of Washington; the treatment of conical cornea by the actual cautery needle, by Dr. J. J. Chisolm, of Baltimore, and the exhaustive paper on traumatic fever, by Dr. B. A. Watson, of Jersey City. Among the papers of lesser note, deserving consideration, are two by Dr. Goodwillie, of New York: one on "Thumb-sucking," with an ingenious apparatus for preventing this disagreeable habit on the part of children, and one on arthritis of the temporo-maxillary articulation.

A number of practical papers read at the meeting fully deserve a perusal.

We can hardly say, however, that the contents of the volume do full justice to the ability of the members of the association. The names we have mentioned constitute only a very small portion of those members who by their contributions should enrich the transactions. It is hardly fair to expect a half-dozen men to do the scientific work of the association, and then to complain of the almost entire lack of value of one-half or two-thirds of the contents of the annual volume. If a sufficient number of first-class contributions could be secured every year to fill the volume, the crowd who furnish the padding would soon drop out of line. If then the transactions were less bulky, they would be of better quality.

*Transactions of the Twenty-eighth Annual Meeting of the Medical Society of North Carolina, held at Asheville, May 31, 1881.*

A volume of 128 pages, of about the usual value of State society transactions. The valedictory address, by the President, Dr. R. B. Haywood, was upon the well-worn subject of the inefficient preliminary education of medical students in this country.

*Enfermedades del Seno Maxilar* (*Cueva de Highmoro*). Por Dr. D. Manuel Antonio Aguilera. Habana, 1881. Pp. 51.

A very complete essay on the diseases of the antrum of Highmore, read before the *Sociedad Odontologica* of Havana, Cuba, on February 26, and March 5, 1881.

*Anales de la Sociedad Odontologica de la Habana,* June to August, 1881, is a monthly publication, giving an account of the proceedings of the Odontological Society of Havana. The membership of the society is small, but active, as will be apparent when we state that the *Anales* contain twenty-four pages monthly, devoted principally to the transactions of the society. A considerable number of dental societies in this country that we know of would do well to imitate their Cuban brethren in the quality of their work.

